# Long-term Survival in a Patient with Butterfly Glioblastoma: A Case Report

**DOI:** 10.7759/cureus.6914

**Published:** 2020-02-07

**Authors:** Megan Finneran, Dario A Marotta, Dana Altenburger, Emilio Nardone

**Affiliations:** 1 Neurosurgery, Advocate BroMenn Medical Center, Normal, USA; 2 Department of Research, Alabama College of Osteopathic Medicine, Dothan, USA; 3 Pathology, Advocate BroMenn Medical Center, Normal, USA; 4 Neurosurgery, Central Illinois Neurosciences Foundation, Bloomington, USA

**Keywords:** glioblastoma, butterfly, bgbm, long-term survival

## Abstract

Butterfly glioblastoma (bGBM) is a malignant glioma that crosses the corpus callous with bilateral cerebral hemisphere involvement. Literature reports are scarce and highlight a dismal prognosis with limited successful treatment options. We describe a patient who survived more than five years from the initial diagnosis. A 44-year-old woman presented to the emergency room for evaluation one day after a motor vehicle collision at the insistence of her husband, with four weeks of confusion, behavioral changes, and increased fatigue. Magnetic resonance imaging (MRI) of the brain revealed an enhancing, heterogeneous mass with significant necrosis, centered in the septum pellucidum and corpus callosum with intraventricular extension. She underwent a stereotactic biopsy of the lesion. Pathology was consistent with glioblastoma, WHO grade IV. She underwent standard radiation treatment and adjuvant temozolomide, demonstrating a near-complete disappearance of the tumor on imaging for the subsequent two years. Upon recurrence, she underwent additional chemotherapy with limited response. A repeat biopsy was positive for a *BRAF* mutation and she was treated with lomustine. After two cycles, she developed thrombocytopenia and shortly after elected to discontinue treatment. She succumbed to the progression of disease five years and two months after the initial presentation. bGBMs are uncommon and highly aggressive brain tumors. A tailored treatment protocol may improve survival. This case marks an unusually long survival of a patient with bGBM and may prompt further research to better understand the behavior of these tumors and how to improve treatment response and survival.

## Introduction

Butterfly glioblastoma (bGBM) is an aggressive form of glioblastoma with bilateral involvement, crossing the corpus callosum to form a butterfly-like tumor [1-3]. Limited literature reports highlight a dismal prognosis with few effective treatment options [4-8]. We describe a unique case of a woman presenting with headaches and behavioral changes, who was found to have a bGBM and had nearly complete resolution of her tumor for two years following a course of radiation and adjuvant chemotherapy. She ultimately succumbed to disease progression five years and two months after diagnosis. Here we discuss this unique case and a relevant literature review of bGBM.

## Case presentation

History

A 44-year-old woman with no past medical history presented to the emergency room at the insistence of her husband one day after a low impact motor vehicle collision. Airbags did not deploy; there was no loss of consciousness and the patient was ambulatory at the scene. The patient complained of a frontal headache, which was unchanged from daily headaches over the previous five months that a primary care provider had attributed to sinusitis. Her husband also reported aggressiveness, short temper, and increased sleeping over the previous four weeks. She drank alcohol socially and did not smoke cigarettes. She had remote family history of brain tumors. Both parents were alive and healthy. 

Examination

Vitals signs are temperature 36.2ºC, blood pressure 119/62 mm Hg, pulse 57/min, respiration rate 16/min, oxygen saturation 99%, and body mass index 22.6 kg/m^2^. The patient was in no acute distress and cooperative during the examination. She was alert and oriented to self, time, and place with no focal neurological deficits. Vision was grossly intact with mild papilledema on the fundoscopic exam. She displayed aggressive behavior, arguing with her husband. The Karnofsky Performance Status was 90.

Diagnostic imaging

**Figure 1:**
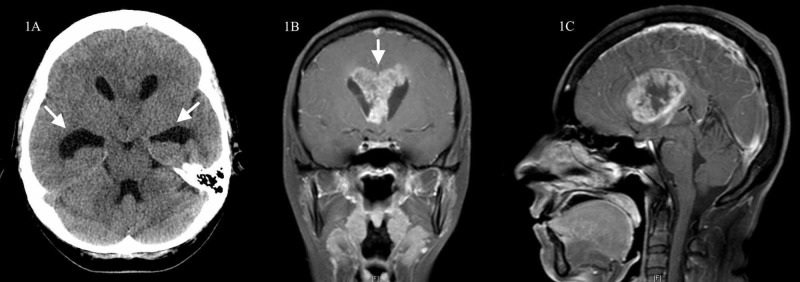
CT head without contrast and post-contrast T1-weight MRI brain showing hydrocephalus with an associated enhancing midline mass CT head without contrast shows hydrocephalus with remarkable dilation of the temporal horns from a midline structure obstructing the foramina of Monro (Figure 1A, left). Post-contrast coronal and sagittal T1-weighted image MRI of the brain shows an enhancing mass involving the septum pellucidum and corpus callosum with a necrotic component (Figures 1B, center; 1C, right). CT, computed tomography; MRI, magnetic resonance imaging.

Computed tomography (CT) head without contrast demonstrated moderate obstructive hydrocephalus with mass effect between the frontal horns of the lateral ventricles at the level of the foramen of Monro, suggestive of an obstructive mass (Figure 1A). Magnetic resonance imaging (MRI) brain demonstrated an avidly enhancing, heterogeneous mass with significant necrosis, centered in the septum pellucidum (Figures 1B, 1C). The mass measured 4.5 cm x 4.0 cm x 4.0 cm. There was an indication of intraventricular extension with subependymal enhancement and transependymal flow along the trigone and occipital horns of the lateral ventricles.

 

Hospital course 

The patient was admitted to the intensive care unit with frequent neurological evaluations and started on dexamethasone. The next day, she underwent right frontal burr hole for stereotactic-guided biopsy. Frozen section suggested a high-grade glioma. She remained neurologically intact and was discharged home on post-operative day number two on a dexamethasone taper.

Pathology

**Figure 2:**
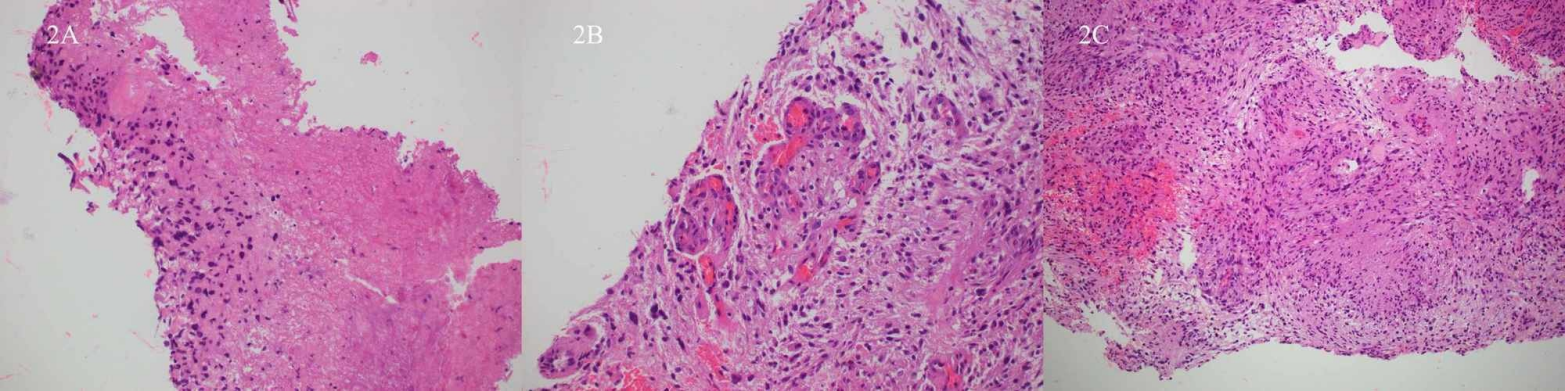
Hematoxylin and eosin stain from brain biopsy consistent with glioblastoma, grade IV Hematoxylin and eosin stain from brain biopsy performed the day after initial presentation show hypercellular glial tissue with pseudopalisading necrosis (Figure 2A, left) and microvascular proliferation (Figure 2B, center). The compilation of nuclear pleomorphism, nuclear atypia, microvascular proliferation, and necrosis (Figure 2C, right) are consistent with glioblastoma, grade IV.

Biopsy showed hypercellular glial tissue with pseudopalisading necrosis, microvascular proliferation, nuclear pleomorphism, and nuclear atypia (Figures 2A-C) consistent with glioblastoma, WHO Grade IV. *IDH1* and *IDH2* were negative. MGMT promoter methylation was present. 

Treatment and outcome

**Figure 3:**
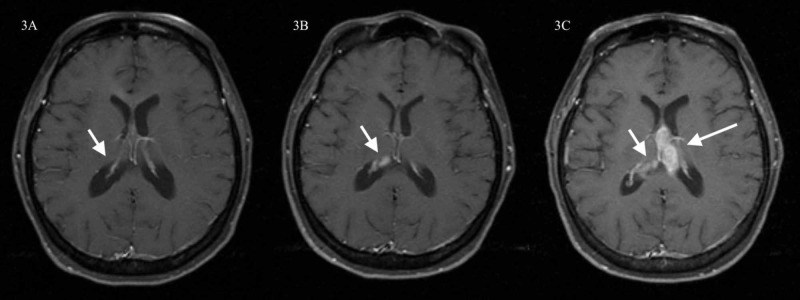
Post-contrast axial T1-weighted image MRI of the brain at two (Figure 3A, left), three (Figure 3B, center), and nearly five years (Figure 3C, right) from the time of diagnosis Post-contrast axial T1-weighted image MRI of the brain shows significant improvement of the lesion with almost disappearance of the periventricular enhancement two years after diagnosis, one year after completion of twelve rounds of temozolomide and six weeks of radiation. The hydrocephalus has resolved (Figure 3A, left). On brain MRI performed the following year, three years after diagnosis, a bilobed focus of enhancement is seen within the right ventricle. This represented the first clear imaging evidence of tumor recurrence (Figure 3B, center). Eighteen months later, brain MRI showed evident progression of the lesion (Figure 3C, right). At that time the patient declined further treatment. She died six months later, more than five years from the original diagnosis. MRI, magnetic resonance imaging.

The patient was treated with radiation therapy for six weeks (30 cycles of 200 cGy), followed by maintenance temozolomide 200 mg/m^2^ days one through five every four weeks for 12 cycles. By completion of temozolomide, the tumor had shown remarkable regression. One year after treatment, there remained little evidence of abnormal MRI signal in the area of the neoplastic lesion with complete resolution of the hydrocephalus (Figure 3A). She noted improvement of her headaches and normalization of her aberrant behavior. 

She underwent routine MRI brain every three months. More than 25 months after her last dose of temozolomide, two foci of enhancement were noted in the right ventricle (Figure 3B). Brain MRIs two and four months later clearly demonstrated recurrence of the tumor. She completed two rounds of temozolomide with no decrease in tumor size. A repeat biopsy for genetic analysis was positive for *BRAF*. After two months of lomustine, she developed thrombocytopenia. She was offered BRAF and MEK inhibitors but declined therapy.

Routine MRI scans remained stable for nine months without intervention; at that time MRI demonstrated remarkable local progression of the tumor (Figure 3C). She underwent no further treatment. She died six months later from progression of her brain tumor, more than five years from diagnosis.

Of note, the patient remained active and functionally independent until the last months of her life. She had an extensive network of support and exhibited an attitude of battling until the last moment.

## Discussion

Glioblastoma (GBM) is an aggressive primary malignancy of the central nervous system, with an incidence of approximately three in 100,000 people per year in the United States [1]. It is associated with rapid progression, with an average survival of 15 months with maximum treatment [1-3]. In roughly 3% of GBM patients, the tumor crosses midline through the corpus callosum, extending inter-hemispherically resembling the wings of a butterfly. The few cases of bGBM documented in the literature describe patients who succumb to the disease in weeks to months, with a median survival of only three months and a six-month survival rate of only 38% [4-8]. Due to the paucity of literature regarding these tumors, prognostic factors specific to bGBM are lacking.

Treatment response of GBM, and presumably bGBM, is closely tied with *IDH* mutations and O6-methylguanine-DNA methyltransferase (MGMT) promoter methylation status. IDH facilitates energy production and when mutated, may deplete reactive oxygen species seeking byproducts, thereby potentiating the efficacy of chemoradiotherapy [9]. MGMT is a DNA repair protein that functions as a dealkylating agent. When the MGMT promoter is methylated, or silenced, cells become more sensitive to alkylating agents used to facilitate apoptosis, such as temozolomide [10]. Of long-term GBM survivors, 95% have MGMT methylated promoters [11], demonstrating its role in prognostication.

bGBM patients with surgical resection experience modestly improved survival compared to those without resection, averaging seven months and 3.5 months, respectively [12]. However, the infiltrative behavior of GBM makes it nearly impossible to eradicate. The bilateral extension of bGBM, in particular, poses a challenge to surgical resection. When weighing the diminished efficacy of resection with the rapid decline of bGBM patients despite treatment, many advocate for needle biopsy with subsequent adjuvant treatment.

One often-overlooked variable of overall survival may be psychosocial stress. The link between depression and terminal illness is well-established, particularly in patients with primary brain cancer [13-15]. The development of depression may, in turn, affect outcomes through the release of epinephrine, norepinephrine, and glucocorticoids [16]. Chronic release of stress hormones can increase immunosuppression that inhibits the anti-tumor response, thus allowing uncontrolled tumor growth [17]. Future research may explore the interplay between psychosocial factors and survival in bGBM.

## Conclusions

bGBM is a devastating diagnosis that poses significant challenges for treatment. Its bilateral extension often precludes surgical resection, yet response to other therapies is limited. Prognosis is poor and survival is cited as weeks to months. Literature reports are uncommon, creating a gap in knowledge regarding management of these aggressive tumors and factors that contribute to survival. Here we discuss a 44-year-old woman with no medical history presenting with headaches and confusion who was found to have a bGBM. Her survival of more than five years is unparalleled and may offer a promising future for similar responses.
